# Biomedical Diagnosis of Leukemia Using a Deep Learner Classifier

**DOI:** 10.1155/2022/1568375

**Published:** 2022-08-29

**Authors:** Tawfeeq Shawly, Ahmed A. Alsheikhy

**Affiliations:** ^1^Electrical Engineering Department, Faculty of Engineering at Rabigh, King Abdulaziz University, Jeddah, Saudi Arabia; ^2^Electrical Engineering Department, College of Engineering, Northern Border University, Arar, Saudi Arabia

## Abstract

Leukemia cancer is the most common type of cancer that occurs in childhood. The most common types are acute lymphocytic leukemia (ALL) and acute myelogenous leukemia (AML) which affect children and adults, respectively. Several health issues occur due to these cancers. Leukemia affects the bone marrow or the lymph nodes. Leukemia produces abnormal white blood cells via the bone marrow system. The affected white blood cells are unable to perform their tasks properly. Detecting leukemia usually requires taking a blood smear from a patient and working with expert hematologists who analyze the smear with a microscope. In this paper, a method to detect ALL and AML using a deep learner classifier is developed and proposed. The method detects both types, determines their severity, and creates a message that recommends next steps to patients. This approach works based on image segmentation and a convolutional neural network (CNN) tool called AlexNet. The obtained results from the proposed approach and using MATLAB reached more than 98% accuracy. The margin exists because several operations are needed to fully detect the blood cancer. A dataset of leukemia from the Kaggle site is used to test the developed method and illustrate its effectiveness. This dataset is C-NMC_Leukemia, and it consists of nearly 10 GB worth of 15,000 images. A confusion matrix of testing images is provided to prove the correctness of the presented approach. Furthermore, a comparative analysis between the proposed algorithm and some works from the literature is presented. This analysis compares the method used to extract features, the classifier that is utilized, the accuracy, the precision, and the recall. The obtained results indicate that the proposed method outperforms other works and produces better results.

## 1. Introduction

The World Health Organization (WHO) reported that around 19 million patients were diagnosed with cancer in 2020 [1–3]. Among them, 10 million patients died. *Cancer* is the most common cause of death since it is aggressive, and its treatments can be complicated and costly [2] [4-5]. Treatment depends on the type of the diagnosed cancer [2–4].

Leukemia is an abnormality that occurs in the blood cells [2]. Blood is responsible for delivering oxygen and nutrients to the cells inside the human body. Furthermore, it transports the produced waste from them [3, 4]. Two types of leukemia have been detected and identified: acute and chronic [2, 5–7]. The first type, acute, is the most dangerous and aggressive since it spreads rapidly, and its symptoms are more severely painful than the second type [2]. The most common types are acute lymphocytic leukemia (ALL) and acute myelogenous leukemia (AML) which affect children and adults, respectively. ALL influences the white blood cells [2, 6–8]. This effect creates an unnecessary number of variations on the white blood cells. ALL occurs in children of age between 3 and 7 [2], and nearly two-thirds of diagnosed reported cases have occurred before age of 6 [2]. WHO claims that leukemia is the primary source of high death rates from cancer [3]. Chronic leukemia grows slower than acute leukemia. Acute leukemia occurs when most of the cells cannot perform their functions, whereas chronic leukemia happens when the normal cells perform their duties while some cells are immature. This situation becomes more threatening as time passes, but there is also a longer window for detection. In cases of acute leukemia, quick detection is critically important.

Blood is composed of red blood cells, white blood cells, and platelets. The red cells carry oxygen to supply the entire organ system, while the white cells protect the body from infections that occur from viruses or bacteria. The platelets support the blood clot process. When leukemia occurs, the body generates more white cells that affect other organs.

**Figure 1:**
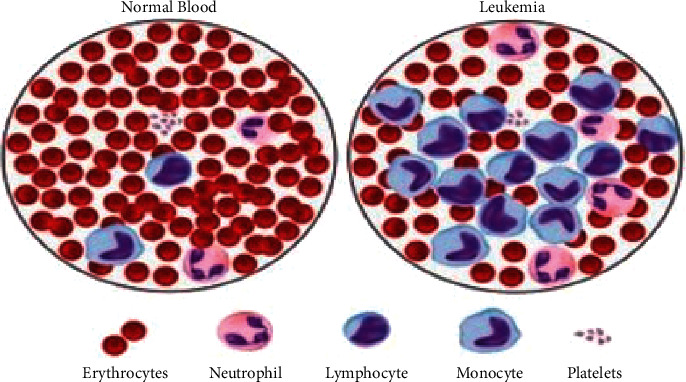
Normal blood vs leukemia blood cells.

These blood abnormalities are detected via a blood smear or sample analyzed with a microscopic machine. Hematologists are key in identifying and classifying leukemia since this process depends on their experience [2, 7–10]. Numerous methods and technologies have been developed and proposed to assist those physicians in detecting and classifying both forms of leukemia. Among these approaches, pattern recognition is widely used in computer-aided systems along with image processing [2]. The time-consuming morphological process is critical in classifying blood cells, and undertaking it requires special skills.  Figure 1 illustrates normal blood cells versus leukemia blood cells.

Segmentation of blood cells is vital to indicating whether blood is healthy or not [5]. Leukemia refers to diseases where some cells are divided without control and cause harm to other tissues [5, 8–11]. Hematologists focus on white blood cells since numerous infections are distinguished by those cells [5–12]. Hematologists classify leukemia as the unusual development of white platelets as depicted in Figure 1 [5]. Diagnosis of leukemia is performed as particular symptoms and signs develop in a patient, which may include frequent infections, losing unplanned weight, and weakness [5]. In addition, fever, pain in the bones, vomiting, and night sweats are symptoms of leukemia, and patients need to pay attention if one or more of these signs occur.

Acute lymphocytic leukemia (ALL), acute myelogenous leukemia (AML), chronic lymphocytic leukemia (CLL), and chronic myelogenous leukemia (CML) are the main four types of leukemia determined and identified by physicians and researchers. ALL starts in the bone marrow, and it is the most common type in children. AML is the most common type in adults. Since ALL and AML are the most common types, this research herein focuses on implementing a fast and reliable algorithm to detect them accurately and precisely.

Researchers and physical physicians have tried to determine the real cause of leukemia with no luck. Numerous factors can trigger leukemia, including radiation exposure and family history of the disease.

Physicians and other healthcare providers can be notified about a possible diagnosis of leukemia by the results of routine blood tests, but additional procedures may be required to have an accurate diagnosis including physical exams, complete blood count (CBC) tests, spinal taps, bone marrow biopsies, and imaging tests, such as CTs and MRIs. Once a patient is diagnosed with leukemia, then their healthcare providers or physicians perform treatments. These treatments may include chemotherapy, radiation, surgery, and biological therapy.

Researchers have developed and proposed numerous systems and approaches to assist physicians and hematologists in diagnosis to achieve higher accuracy [5, 11, 14]. These systems can be used to speed up the diagnosis of leukemia [6]. The highly efficient method to diagnose leukemia uses convolutional neural networks (CNNs) [5–7]. CNNs are difficult to deploy since they are associated with a high computational cost [19, 20].

This paper proposes a method to detect AAL and AML efficiently. It develops and proposes a feasible and reliable method to detect leukemia in real time while maintaining high accuracy. This approach detects AAL and AML based on the convolutional neural network (CNN) and image segmentation using MATLAB.

The remainder of this paper is organized as follows: a literature review is presented in Section 2, and Section 3 provides details about the developing approach. Discussion and results are provided in Section 4, and the conclusion is given in Section 5.

Mondal et al. in [1] used CNNs to automate the detection of AAL from microscopic images. The authors recommended a classifier based on the weighted ensemble of different deep CNNs. Accuracy, *F*1-score, and kappa values were the performance metrics that were evaluated according to the weighted ensemble method. The obtained accuracy was nearly 86%, and the *F*1-score was approximately 89%. In this paper, for comparison, the achieved accuracy is nearly 94% and it can detect both types of leukemia at an early stage.

Oliveira and Dantas in [2] proposed a simple alteration to standard neural network (NN) construction to reach higher performance in the classification problem of the malignant leukocyte. Three constructions were tested to verify the proposed approach. In addition, around 93% of the *F*1-score was achieved when tested on the three constructions. Several metrics were evaluated, namely, accuracy, precision, sensitivity, specificity, and *F*1-score. In this proposed method, accuracy, the number of defected blood cells, and the percentage of cancer are the metrics that are considered. The developed approach can also detect ALL and AML with accuracy over 97%. Interested readers can find more information in [2].

In [3], Shaheen et al. developed a model to classify and detect AML in microscopic images based on the AlexNet approach. The authors claimed that their model reached 89% accuracy and nearly 88% precision on a dataset that contained 4 thousand blood smears. A comparison study between AlexNet and LeNet was conducted, and it showed that there was a slight difference between both models in which AlexNet performed better than LeNet. More information can be found in [3]. Herein, the proposed approach reaches almost 98% of accuracy for AAL and AML, while the method in [3] could only identify AML.

Sashank et al. in [6] proposed two different classification methods to detect AAL using deep learning techniques. An ALL-IDB2 dataset was utilized, and it contained microscopic images of blood samples. The authors used AlexNet and a machine learning model to detect ALL. CNNs, SVM, KNN, XGBoost, and decision tree were utilized as well. The obtained results from the second approach were better than those from the first approach, and the highest obtained accuracy was 100% in classification as reported by the authors. The used dataset contained 760 lymphocyte images, and from these, 570 images were used for training while the rest were used for testing. Readers can get more information in [6].

Claro et al. in [7] presented a CNN architecture to differentiate blood slides that contained ALL, AML, and healthy blood slides (HBS). In [7], 16 datasets were utilized to conduct several experiments. These datasets contained 2415 images, and the method obtained 97% accuracy and precision. The authors performed a comparison experiment with numerous methods that used CNNs only. More information in [7] can be obtained for interested readers.

In [9], Dasariraju et al. presented a method to detect and classify AML using a machine learning model based on analyzing immature leukocytes. The authors obtained their dataset from the *Cancer* Imaging Archive, which contained data from AML patients and healthy patients. The authors used image format conversion, multi-Otsu threshold, and morphological process. In addition, 16 features were extracted from every image. A random forest algorithm (RFA) was used to train the dataset, and it produced nearly 93% of accuracy in detection and almost 94% in classification. However, the obtained precision only reached 65%, far less than the model in this paper. The developed model in this paper also detects AAL and AML with an accuracy over of 98% exceeding the presented model in [9].

Pallegama et al. in [10] proposed a method to detect ALL cells using CNNs. The authors claimed that their approach could reduce the time needed for analyzing the blood samples and the cost for microscopic observations. Over 100 blood smears were used to train the method to detect ALL cells. These blood samples were diagnosed ALL by a cancer hospital. The proposed approach herein detects ALL and AML, so it is better than method that was presented in [10].

Loey et al. in [11] presented two automation methods based on a transfer learning approach to detect leukemia. In the first method, a pretrained AlexNet was used to extract features from blood microscopic images. In the second method, fine-tuning was performed for all extracted features to detect leukemia. Both methods were tested on a dataset that contained around 3000 images. The second approach performed better than the first one in classification and claims 100% accuracy. Interested readers can refer to [11] for more information.

Bhandari et al. in [21] performed a comprehensive analysis of the state-of-the-art methods to detect cancer utilizing genetic algorithms. The authors made a deep analysis to identify the future challenges in the development of such techniques. This analysis was related to various types of cancer such as bladder, breast, ovarian, and leukemia. The authors focused on the type of cancer, functions being used, the main purpose of the methods, and the type of data being tested and verified. Additional information can be in [21].

In [22], Hamza et al. implemented a method to detect and classify ALL using an optimal deep transfer learning method. Blood smear images were utilized for detection and classification purposes. A filter was used to remove noise, and the fuzzy c-means method was involved to segment the inputs. Features were extracted using the competitive swarm optimization and NetB0 approaches. The authors measured several performance metrics including accuracy, precision, recall, specificity, and F-score. The authors claimed that their algorithm achieved 96%, 95.715%, and 96.51% accuracy, precision, and recall, respectively. Even so, our approach detects and classifies ALL and AML with higher accuracy, precision, and recall. These results indicate that the proposed algorithm herein outperforms the implemented method in [22].

Abir et al. in [23] developed a method to detect ALL using a transfer learning model. This method achieved nearly 98.3% of accuracy, while our presented algorithm achieves nearly over 99% of accuracy. Four different types of models were utilized. However, these four models detected only ALL, while our algorithm detects and classifies ALL and AML as well. Additional information is found in [23].

## 2. Materials and Methods

**Figure 2:**
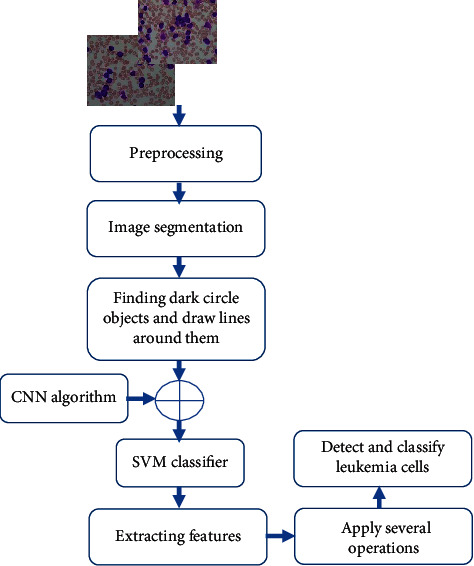
Flowchart of the proposed algorithm.

The proposed approach began with a patient who suffered greatly from leukemia. He was diagnosed with leukemia when he was 6 years old. Initially, his physician diagnosed him with an infection, and the leukemia was only detected later. Due to his advanced condition, he had to go through a complex treatment, but the cost was too high. It became critical to consider a new method for leukemia detection. The proposed method detects ALL and AML since both types occur more than others. This paper uses an 8-layer CNN called AlexNet. All images used are 227 × 227 pixels in size. MATLAB is used as a programming platform and a simulation tool to train the developed approach. The proposed algorithm contains several processes as depicted in Figure 2.

The proposed approach is illustrated in the following Algorithm 1:

AlexNet is involved to extract features of the white blood cells to determine healthy and infected cells. The presented algorithm learns itself regularly according to the obtained results. The extracted features include mean squared error (MSE), histogram of oriented gradients (HOG), and local binary pattern (LBP). In addition, other features are extracted and utilized as well.

One dataset was used to train, validate, and test the proposed algorithm. In addition, some metrics are evaluated during the simulation including the following:

(1) True Positive (TP): it measures a total number of correctly identified blood samples to detect leukemia ALL and AML.
(2) False Positive (FP): it refers to incorrectly identified samples.
(3) True Negative (TN): it defines a number of total negative samples that were detected and classified correctly by the algorithm.
(4) False Negative (FN): it provides an indication of a total number of the negative samples that were incorrectly classified and detected.
(5) Precision (PRE): this metric measures a fraction of the true samples that are identified correctly over the same samples as well as the samples that are classified incorrectly as shown in
  (1) $$\begin{array}{c} \text{PRE}=\frac{\text{TP}}{\left(\text{TP}+\text{FP}\right)}. \end{array}$$
(6) Recall (REC): it indicates the fraction of the truly identified samples over the summation of true samples plus the number of negative samples that are classified incorrectly as depicted in
  (2) $$\begin{array}{c} \text{REC}=\frac{\text{TP}}{\left(\text{TP}+\text{FN}\right)}. \end{array}$$
(7) Accuracy: this metric shows the percentage of the summation of the true samples and the negative ones that are detected and classified correctly over the total number of samples as depicted in
  (3) $$\begin{array}{c} \text{Accuracy}=\frac{\left(\text{TP}+\text{FN}\right)}{\left(\text{TP}+\text{TN}+\text{FN}+\text{FP}\right)}. \end{array}$$

**Figure 3:**
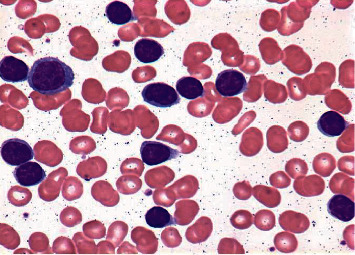
The original sample of AAL.

**Figure 4:**
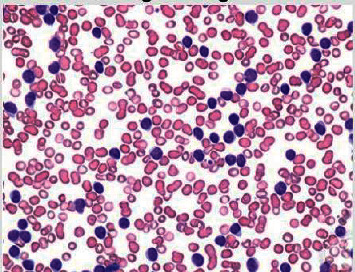
The original sample of AML.

As stated earlier, the proposed method uses one dataset in which around 10,500 blood samples, 70% of the dataset, are assigned for the training purpose. The remaining 30% of the dataset is divided into two groups: 15% for testing and 15% for validating the results. For the validation, there are 2,250 images of blood samples. Figures 3 and 4 illustrate the original images of ALL and AML, respectively. In the training stage, the samples are either healthy or infected. For every input or sequence of inputs, the presented algorithm extracts feature from healthy samples and infected samples as well. These features are deeply analyzed in order for the implemented model to be able to determine and classify ALL and AML accurately.

**Figure 5:**
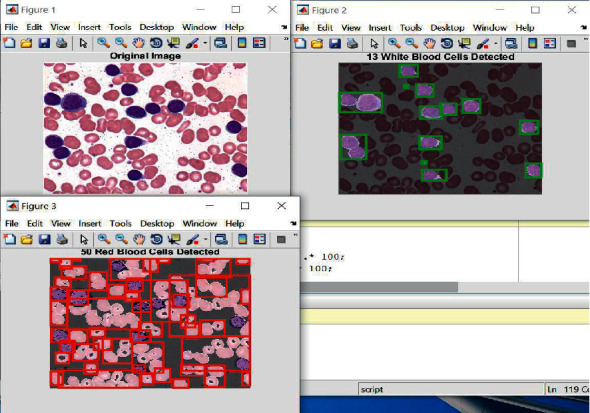
Sample outputs from the developed method.

Figure 5 illustrates the obtained outputs from the presented approach in which white and red blood cells detected in a random blood sample are surrounded by green and red rectangles, respectively. The upper left image shows the original image, the upper right picture represents the resultant image of the detected white cells, while the bottom left picture denotes the detected red blood cells of the sample image.

The proposed algorithm identifies the white and the red blood cells in the input images as shown in Figure 5. The white blood cells are counted and surrounded by green rectangles, while red rectangles are drawn around every red cell, as illustrated in Figure 5. These results are utilized later in the deep learning and classification phases.

## 3. Results and Discussion

MATLAB is used to conduct several experiments to process images of blood samples that are either healthy or infected. The infected samples are infected by ALL and AML. To detect and classify AML or ALL using the presented algorithm, 10,500 images of blood smears are used to train the algorithm in detection and classification, while 2,500 images are utilized for validation. The remaining images are used and utilized for testing. Support vector machine (SVM) performs the classification operation. The 2,500 of testing images are also used to evaluate accuracy, precision, and recall.

**Example 1 .**

Leukemia: AML cells.

**Figure 6:**
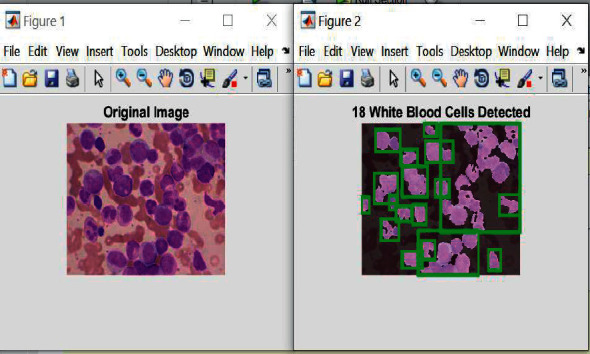
Detected white blood cells.

**Figure 7:**
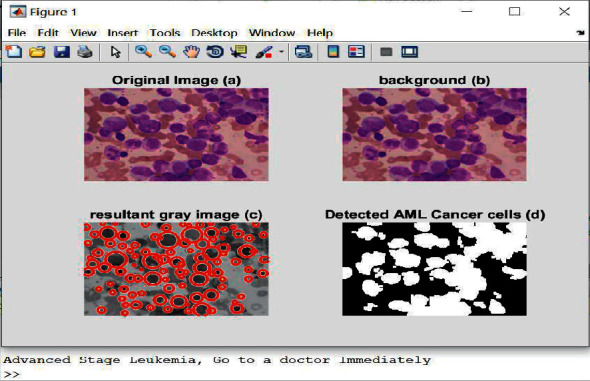
The obtained outputs of AML leukemia.

Figure 6 displays the detected white blood cells of AML type. These cells are surrounded by the green rectangles.  Figure 7 illustrates the obtained results of the detection and classification operations.

Figure 7(a) represents the original image, 7(b) shows the original image after removing noises, while 7(d) highlights the detected AML cancerous cells in white color.

**Example 2 .**

Leukemia: ALL cells.

**Figure 8:**
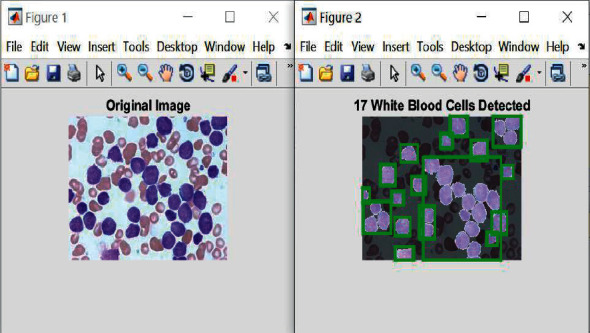
Obtained results for detecting white blood cells affected by ALL.

**Figure 9:**
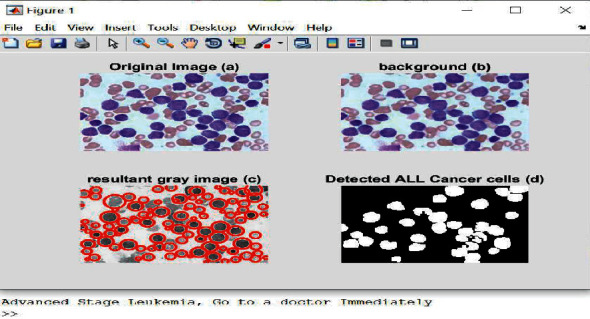
The obtained results of ALL leukemia detection.

Figure 8 shows the detected white blood cells of ALL enclosed by the green rectangles, and Figure 9 demonstrates the obtained results along with a message to the patient.

Table 1 lists the values of all mentioned metrics that were determined by the developed approach.

The proposed approach detects both types of leukemia with an accuracy of over 98% as shown in Table 1.  Table 2 depicts the confusion results on the testing dataset represented in a confusion matrix. The corrected identified results are distinguished in green, while red boxes refer to the inaccurate detection and classification of ALL and AML, respectively.

The comparison study between the presented algorithm and other developed approaches in the literature is conducted and shown in Table 3. This comparison evaluates three-performance metrics which are precision, recall, and accuracy. All results are given in Table 3. This table lists the works that were developed with their references' numbers, the methodologies used to extract features, their classifiers, and the three-performance metrics for comparison. The values for all metrics are the overall obtained results.

Table 3 shows that the proposed algorithm herein outperforms and outstands most of the developed and implemented approaches in the literature. This indicates that the developed approach in this research produces promising results.

## 4. Conclusions

The proposed method has the capability to detect and classify ALL and AML cancer with high precision and accuracy as proved by the conducted experiments. Hence, it can be used in hospitals and healthcare centers to support and assist hematologists and laboratory technicians in their tasks. In addition, the developed algorithm reaches an accuracy of nearly 99% in detection and classification.

## Figures and Tables

**Algorithm 1:**
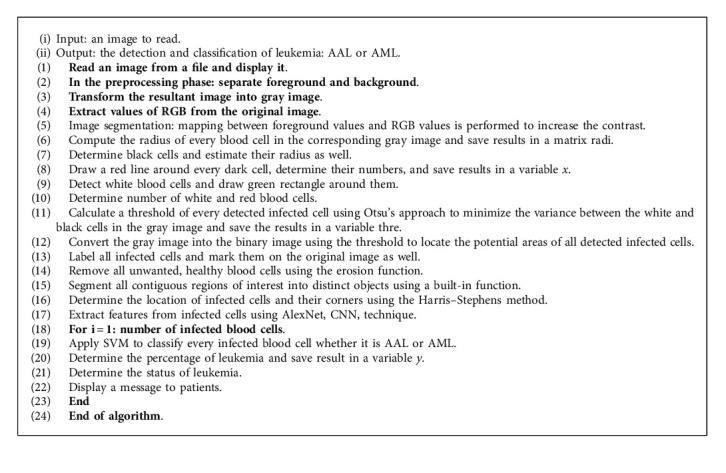
Leukemia detection and classification.

**Table 1:** Evaluated performance metrics.

Number of testing images: 2,500.	
Metric	Evaluated value
TP	1981
FP	14
TN	494
FN	11
PRE	99.30%
REC	99.45%
Accuracy	99%

**Table 2:** Confusion matrix of the testing dataset.

Predicted Class	True class		
		ALL	AML
	ALL	953 = (98.55%)	14 = (1.45%)
	AML	11 = (1.08)	1002 = (98.92%)

**Table 3:** Comparison of three-performance metrics.

Developed method	Method to extract features	Classifier name	Accuracy	Precision	Recall (%)
Mondal et al., 2021 [1]	CNN	CNN	86.2%	88.7%	88.8
Oliveira and Dantas, 2021 [2]	CNN	CNN	91.49%	89.61%	93.90
Shaheen et al., 2021 [3]	CNN	AlexNet	98.58%	87.4%	88.9
Sashank et al., 2021 [6]	CNN	SVM, KNN, decision trees	95.05%	95.25	96.75
Claro et al., 2020 [7]	CNN	CNN	97.18%	97.23%	97.18
Dasariraji et al., 2020 [9]	Random forest	Random forest	92.99%	91.23%	95.41
Loey et al., 2020 [11]	CNN	FC	99.04	99.64%	98.44
The proposed method	CNN (AlexNet) + SVM	SVM	99.30%	99.45%	99

## Data Availability

The authors would like to confirm that the dataset which is utilized in this research is available at the Kaggle website and can be found at the following link: https://www.kaggle.com/datasets/andrewmvd/leukemia-classification.
